# Elevated FOXA1 Expression Indicates Poor Prognosis in Liver Cancer due to Its Effects on Cell Proliferation and Metastasis

**DOI:** 10.1155/2022/3317315

**Published:** 2022-08-05

**Authors:** Zhenrong Liu, Yaru Wang, Zulihumaer Aizimuaji, Sheng Ma, Ting Xiao

**Affiliations:** State Key Laboratory of Molecular Oncology, Department of Etiology and Carcinogenesis, National Cancer Center/National Clinical Research Center for Cancer/Cancer Hospital, Chinese Academy of Medical Sciences and Peking Union Medical College, Beijing 100021, China

## Abstract

**Purpose:**

Studying the pathogenesis of liver cancer is conducive to the exploration of effective diagnostic and prognostic biomarkers. In this study, we investigated the expression of FOXA1 and its oncogenic role in hepatocellular carcinoma (HCC).

**Methods:**

Transcriptome data of HCC tissues were downloaded from The Cancer Genome Atlas (TCGA) and GEO databases and analyzed using R software. We also upregulated FOXA1 expression in HCC cells and investigated the role of FOXA1 in the proliferation and migration of HCC cells through proliferation, colony formation, wound healing, and Transwell assays.

**Results:**

An analysis of the transcriptome data in TCGA database revealed found that FOXA1 is highly expressed in HCC tissues and that patients with low FOXA1 expression have a better prognosis. High FOXA1 expression was mainly associated with extracellular matrix organization, cancer, and mitosis. The results of an immunohistochemistry (IHC) assay showed that FOXA1 protein was highly expressed in HCC tissues, and patients with low FOXA1 expression showed longer disease-specific survival times and progression-free intervals. The results from quantitative reverse transcription–PCR (RT–qPCR) and Western blot experiments showed that the expression of FOXA1 in liver cancer cell lines was higher than that in immortalized human liver cell lines. Proliferation, wound healing, and Transwell experiments showed that FOXA1 enhanced the proliferation and migration abilities of liver cancer and immortalized human cell lines.

**Conclusion:**

Our research suggests that FOXA1 plays an important role in promoting the recurrence and metastasis of HCC by increasing cell proliferation and metastasis.

## 1. Introduction

Liver cancer is a malignant tumor that seriously harms human health, is the sixth most common malignant tumor in the world, and ranks third as the cause of cancer deaths worldwide [1, 2]. Primary liver cancer is mainly classified into hepatocellular carcinoma (HCC) and cholangiocarcinoma (CCA), and HCC accounts for approximately 85-90% of all primary liver cancers. The main etiologies of primary liver cancer include chronic infection with hepatitis B virus or hepatitis C virus, aflatoxin exposure, high alcohol intake, and metabolic diseases [3]. Most patients with liver cancer are in the middle and late stages of the disease at the time of diagnosis, and their prognosis is poor. The five-year survival rate is lower than 5%, which poses a serious threat to people's health [4]. Surgical resection, liver transplantation, chemotherapy, radiotherapy, percutaneous ethanol injection, radiofrequency ablation, and various embolization and molecular targeted therapies are currently applied in the clinical treatment of liver cancer, but these treatments are not ideal, and the mortality rate remains very high [5–7]. Therefore, determining the pathogenesis of HCC and finding new therapeutic targets are urgent needs.

Transcription factors (TFs) are proteins that coordinate gene expression in a spatiotemporal manner in specific cell types. TFs control chromatin and transcription by recognizing specific DNA sequences and form a complex system that guides genome expression [8, 9]. A variety of TFs regulate target genes in a variety of ways. TFs also play crucial roles in tumor growth; cell differentiation, apoptosis, metastasis, and invasion; and drug resistance [10]. Forkhead box A1 (FOXA1) is the first transcription factor belonging to the forkhead box family of proteins [11]. The proteins of the forkhead box family have conserved DNA-binding domains that play important roles in cell cycle regulation, lipid metabolism, and embryonic development [12]. FOXA1, also known as HNF3A, MGC33105, and TCF3A, is composed of 472 amino acid residues, and its forkhead box domain (FHD) sequence is highly conserved and consists of 110 amino acid residues [13]. FOXA1 plays an important role in growth and development and in the occurrence and metastasis of tumors by playing different roles in different tumors [14]. FOXA1 has been widely studied in urinary system tumors and breast cancer [13]. As a transcription factor, FOXA1 can bind to androgen receptor (AR) and is highly expressed at the early stage of prostate cancer. FOXA1 can also regulate the transcription and translation of AR genes and increases androgen synthesis, which results in the promotion of prostate cancer cell metastasis [15]. In addition, FOXA1 expression is correlated with estrogen receptor (ER) positivity in breast cancer cell lines [16]. Some studies have found that FOXA1 plays an important role in the occurrence and development of liver cancer [17], but few studies have recently focused on this topic.

Through database analyses and experimental verification, this study found that FOXA1 is closely related to the clinicopathological indicators and prognosis of HCC patients, and this finding provides a theoretical basis for the clinical application of FOXA1 and suggests that FOXA1 may become a new target for HCC targeted therapy.

## 2. Materials and Methods

### 2.1. Datasets and Sample Collection

The GSE121248 and GSE62232 mRNA expression datasets were downloaded from the Integrated Gene Expression Omnibus (GEO) (http://www.ncbi.nlm.nih.gov/geo), a common repository for data storage [18]. Both datasets were based on the GPL570 platform. The GSE121248 dataset included 70 liver cancer tissues and 37 normal tissues, and the GSE622322 dataset included 81 HCC tissues and 10 normal tissues. The limma package in R software was used to analyze differentially expressed genes (DEGs), and the ClusterProfiler package was used to analyze the Gene Oncology (GO) functions and Kyoto Encyclopedia of Genes and Genomes (KEGG) pathways of the DEGs. Using the STRING database (https://string.db.org), a network of the interactions of differentially expressed proteins was constructed with *P* < 0.01 set as the boundary value, and hub genes were identified and visualized using Cytoscape software.

Transcriptome data from 374 samples of HCC tissues and 50 samples of normal tissues and patients' clinical information were downloaded from The Cancer Genome Atlas database (https://portal.gdc.cancer.gov/). Using the Survminer package surv_cutpoint in R software to determine the cutoff value, the HCC samples were classified into a group with low FOXA1 expression and a group with highFOXA1 expression based on the FOXA1 expression levels. The group with low FOXA1 expression included 125 HCC samples, and the group with high FOXA1 expression included 240 HCC samples. The Survival package was used for prognostic analysis.

HCC tissue chips were purchased from Shanghai Zholi Biotechnology Co., Ltd. and included 49 pairs of HCC and paracancerous tissue samples with complete clinicopathological information and detailed follow-up information.

### 2.2. Main Reagents and Main Instruments

Huh7 cells were grown in DMEM supplemented with 10% fetal bovine serum (Gibco, USA). A mouse monoclonal antibody against FOXA1 (ABclonal, USA, Art.:A15278); an anti-GAPDH mouse monoclonal antibody (ProteinTech, USA, Art. HRP-60004) and a bicinchoninic acid (BCA)^TM^ protein assay kit (Thermo Fisher, USA) were used in this study. Both the pCMV6-FOXA1 vector (Art.: RC206045) and pCMV6-entry vector used in this study were purchased from the OriGene Company in the United States.

A carbon dioxide incubator (Heraeus, Germany), an electrophoresis tank with a wet transfer electrometer (Bio-Rad, USA), a chemiluminescence imaging system (GE, USA), a real-time quantitative PCR kit (ABI, USA), a PCR instrument (Biometra, Germany), and a real-time dynamic living cell monitor (Essen, USA) were used in the experiments.

### 2.3. RNA Extraction and qRT–PCR Analysis

TRIzol reagent was used for the extraction of total cell RNA, and a reverse transcription reagent was used for cDNA synthesis. The TB Green™ Premix Ex Taq™TliRnaseH Plus kit (TaKaRa, Japan, RR420A) was used for PCR with GAPDH (upstream, 5′-TCGGAGTCAACGGATTTGGT-3′, and downstream, 5′-TTCCCGTTCTCAGCCTTGACGAPDH-3′) as an internal reference. The following primers for FOXA1 were used: upstream, 5′-CTACTACgCAGACACGCAGG-3′, and downstream, 5′-TCATGTTGCCGCTCGTAGTC-3′. The 20 *μ*L reaction system was subjected to the following reaction conditions: predenaturation at 95°C for 30 s, 40 cycles of 95°C for 5 s and 60°C for 34 s, 95°C for 15 s, 60°C for 1 min, and 95°C for 15 s. Each experiment was replicated in three wells. The RT–qPCR results were analyzed by the 2^−△△CT^ method. The experiment was repeated three times.

### 2.4. Western Blot Analysis

The cells were digested with 0.5% trypsin-EDTA, centrifuged, precipitated in protein extract (RIPA lysate : cocktail : PMSF = 100 : 1 : 1), lysed on ice for 30 min, and centrifuged. Total protein was extracted from the supernatant. A BCA protein quantitative kit was used for determination of the protein concentration, and a standard curve was drawn. Samples of 30 *μ*g protein per well were loaded onto electrophoresis gels after mixing and denaturation in a metal bath at 100°C for 10 min. Electrophoresis was performed at 80 V for 30 min and 120 V for 1.5 h; the proteins were transferred to membranes at 250 mA for 2 h; the membranes were blocked with 5% skim milk for 2 h and incubated overnight with primary antibodies (anti-FOXA1 mouse monoclonal antibody diluted 1 : 2000 and anti-GAPDH mouse monoclonal antibody diluted 1 : 4000). After washing, the membrane was incubated with secondary antibody (goat anti-rabbit/mouse diluted 1 : 2000 dilution) for 1 h, exposed and imaged.

### 2.5. Proliferation and Colony Formation Assays

Cell lines carrying no-load and FOXA1-overexpressing plasmids were evenly seeded in a 96-well plate at 4000 cells/well, with replicates in 5 wells. The cells were incubated overnight in an incubator at 37°C. The next day, the cells in the two groups were placed in a live-cell-monitoring instrument for observation and photographing. Photographs were taken every 12 h to observe the proliferative ability of the two groups of cells by detecting the degree of cellular convergence. Cell lines carrying no-load and FOXA1-overexpressing plasmids were evenly seeded into 6 cm petri dishes at 2000 cells/dish. The culture was refreshed every 3 days. After 14 days of incubation, the surviving colonies were fixed with precooled methanol and stained with 0.5% crystal violet. The colonies were subsequently counted. Three independent experiments were performed.

### 2.6. Wound Healing and Transwell Migration Assay

Cell lines carrying no-load and FOXA1-overexpressing plasmids were evenly seeded in a 96-well plate with 3 × 10^4^ cells/well, and replicates in three wells were included. The cells were incubated overnight in an incubator at 37°C. On the second day, the cell monolayers were wounded using a scratch device, and the medium was replaced with serum-free medium. The wounded cells were placed in a living cell monitoring instrument for photography and observation. Photographs were taken every 12 h to observe the migration ability of the cells in the two groups by measuring the changes in the width of the wound. The cells (100,000 cells per well) were inoculated into a Transwell compartment containing medium with fetal bovine serum (FBS), and 600 *μ*l of medium containing FBS (10%) was then added laterally to the compartment. After culture at 37°C for 16-48 h, the cells that had migrated to the lower surface of the chamber were fixed and stained with crystal violet. Images of the cells on the membrane were collected under a microscope. Different views were randomly selected for counting, the counts were subjected to statistical analysis, and the average values were obtained.

### 2.7. Immunohistochemistry (IHC)

A tissue microarray with 49 HCC tissues and adjacent paired tissues was placed in an oven and baked at 65°C for 4-5 h. The tissue microarray was dewaxed, hydrated with xylene, and then incubated in a 3% H_2_O_2_ solution at room temperature for 15 min in the dark for the removal of endogenous peroxidase activity. Antigen repair solution was used for antigen repair, and normal goat serum blocking solution was used to seal the samples at room temperature for 20 min. The samples were incubated with primary antibody working solution (anti-FOXA1 mouse monoclonal antibody diluted 1 : 100) overnight at 4°C. The cells were then incubated with the secondary antibody at room temperature for 20 min. The staining intensity of 3,3′-diaminobenzidine (DAB) was observed under a microscope. Hematoxylin-stained cells were sealed with neutral resin and observed under a microscope.

The results were determined as follows: the IHC results were independently evaluated by 2 pathologists from the Cancer Hospital, Chinese Academy of Medical Sciences. Comprehensive scoring was performed based on the percentage of positive cells and the cell staining intensity. The scoring rules used to identify the percentage of positive cells were as follows: 0 for 0-5% positive cells, 1 for 5-25% positive cells, 2 for 25-50% positive cells, 3 for 50-75% positive cells, and 4 for 75-100% positive cells. The scoring rules for the cell staining intensity were as follows: yellow (with no brown), 0; light yellow, 1; brownish yellow, 2; and brown, 3 [19]. The IHC results were then classified into 4 grades as follows: the percentage of positive cells and the cell staining intensity score were multiplied, and resulting scores of 0, 1-4, 4-8, and 8-12 were classified into grades of 0, 1, 2, and 3, respectively. A score of 0 indicated low protein expression, whereas scores of 1, 2, or 3 indicated high protein expression.

### 2.8. Tumor Xenograft Model

NTG mice (female, aged 6 weeks) purchased from Sibafu (Beijing, China) Biotechnology Co., Ltd. are a type of mouse with severe combined immunodeficiency. The mice were randomly divided into two groups (*n* = 6 per group). Huh7 cells (2 × 10^6^ per injection) that were transfected with FOXA1-overexpressing plasmid and empty plasmid were implanted into the right flank of the mice via subcutaneous injection. The tumor volume was measured every 3 days after obvious observation and calculated using the following formula: volume = (length × width^2^)/2. The tumor growth curve was plotted. After 4 weeks, all the mice were sacrificed under anesthesia, and the tumors were removed and weighed. The animal experiment plan was approved by the Animal Ethics Committee of Cancer Hospital of Chinese Academy of Medical Sciences.

### 2.9. Data Analysis

R software was used to analyze the transcriptome data obtained from the GEO and TCGA databases. The grayscale values from the Western blotting assay were analyzed using ImageJ 1.8.0 software. SPSS 23.0 and GraphPad Prism 8.0 were used for the statistical analyses. The relationship between FOXA1 expression and clinicopathological parameters in TCGA database and HCC tissues was analyzed by the *χ*^2^ test. The Kaplan–Meier method was used to plot the survival curve of HCC patients, and the log-rank test was performed. The significance level was set to *α* = 0.05.

## 3. Results

### 3.1. GEO Dataset Screening for Hub Gene Identification

**Figure 1:**
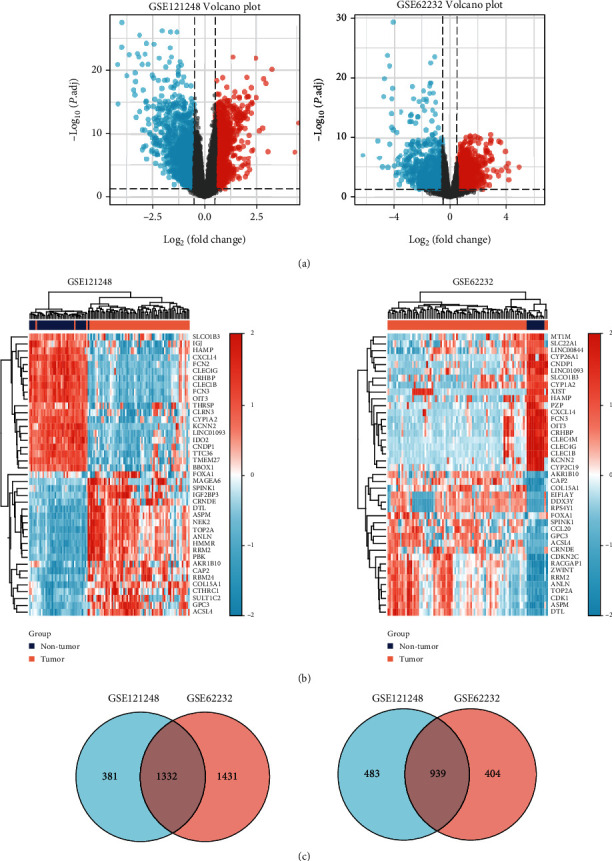
Identification of DEGs in the GEO dataset. (a) Volcano plot of the DEGs in the GSE121248 and GSE62232 datasets. (b) Cluster analysis of the DEGs in these two GEO datasets. (c) Venn plots revealed 2271 common DEGs, including 1332 coupregulated genes (left) and 939 codownregulated genes (right).

**Figure 2:**
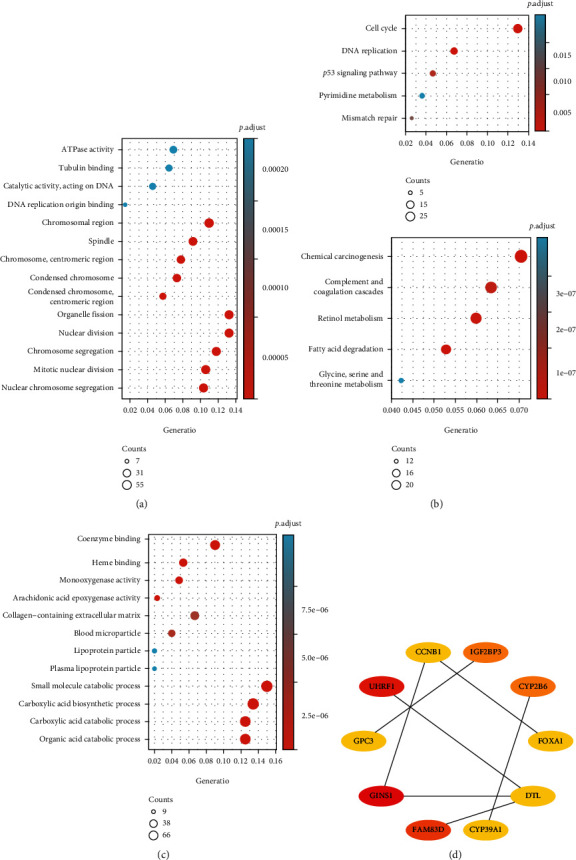
GEO dataset hub genes. (a) Gene Oncology analysis of upregulated intersecting DEGs. (b) Gene Oncology analysis of downregulated intersecting DEGs. (c) KEGG pathway enrichment analysis of upregulated and downregulated intersecting DEGs. (d) Most significant module in the PPI network.

The GSE121248 and GSE62232 datasets were downloaded from the GEO database. The limma package in R software was used to analyze the DEGs in the dataset, and the ClusterProfiler package was used for GO function analysis and KEGG pathway analysis of the DEGs. A total of 3135 and 4106 DEGs were identified in the GSE121248 and GSE62232 datasets, respectively (Figure 1(a)). Among these DEGs, 1713 upregulated genes and 1422 downregulated genes were identified in the GSE121248 dataset, and 2763 upregulated genes and 1343 downregulated genes were identified in the GSE62232 dataset. In addition, 2271 common DEGs, including 1332 coupregulated genes and 939 codownregulated genes, were obtained (Figure 1(c)). A cluster analysis of all the DEGs was performed, and only the first 41 genes with the most significant differences were selected for visualization (Figure 1(b)). The GO function analysis of DEGs showed that the upregulated genes were mainly involved in ATPase activity, tubulin binding, DNA replication origin binding, and other related processes, whereas the downregulated genes were mainly involved in coenzyme binding, heme binding, monooxygenase activity, and other biological processes (Figures 2(a) and 2(b)). The KEGG pathway analysis of the DEGs showed that the upregulated genes were mainly involved in the cell cycle, DNA replication, P53 signaling pathway, and other related pathways, whereas the downregulated genes were mainly involved in chemical carcinogenesis, complement and coagulation cascade, retinol metabolism, and other pathways (Figure 2(c)). A PPI network was constructed using the STRING database and Cytoscape software (Figure 2(d)), and a total of 10 hub genes were identified: FOXA1, CCNB1, IGF2BP3, UHRF1, GPC3, GINS1, FAM83D, CYP39A1, CYP2B6, and DTL. A literature review [20] revealed that FOXA1 is an essential TF in liver development; thus, FOXA1 was selected for further analysis and verification.

### 3.2. TCGA Bioinformatics Analysis

**Figure 3:**
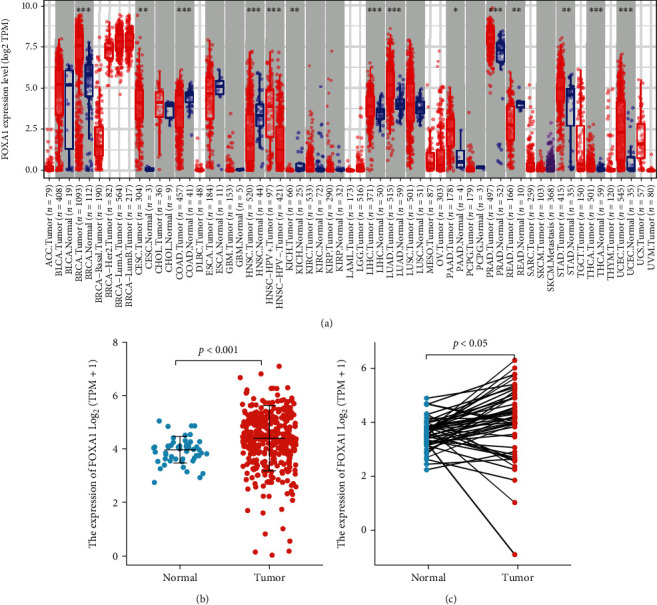
FOXA1 expression in HCC samples in TCGA. (a) The expression of FOXA1 in different cancer species was obtained with the TIMER database. (b) FOXA1 expression in 374 HCC samples and 50 paracancerous tissue samples from TCGA. (c) FOXA1 expression in 50 pairs of HCC samples from TCGA.

**Figure 4:**
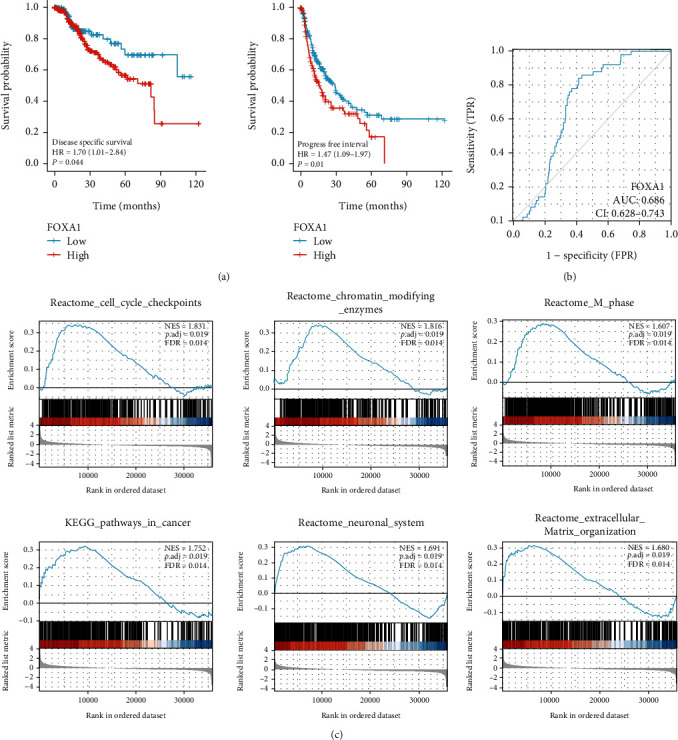
TCGA bioinformatics analysis. (a) The Kaplan–Meier analysis results for disease-specific survival (DSS) and progression-free interval (PFI) were compared between patients with high and low expression of FOXA1. (b) ROC curves of FOXA1. (c) Gene set enrichment analysis (GSEA) of samples from TCGA with high FOXA1 expression and low FOXA1 expression.

We utilized the TIMER database (https://cistrome.shinyapps.io/timer/) to evaluate the difference in FOXA1 expression among various tumor types. The gray background indicates that FOXA1 strikingly differs among cancer types. FOXA1 expression was significantly higher in hepatocellular carcinoma (LIHC), lung adenocarcinoma (LUAD), lung squamous cell carcinoma (LUSC), and cervical squamous cell carcinoma and cervical adenocarcinoma (CESC) than in the normal control group (Figure 3(a)). We obtained 374 HCC transcriptome samples with complete clinical information and 50 paracancerous tissue transcriptome samples from TCGA public database. The FOXA1 expression level was analyzed using R software, and the results showed that the FOXA1 expression level was higher in the HCC tissue samples (*P* < 0.05) (Figures 3(b) and 3(c)). The Survminer package in R software was used to find the cutoff value, and the FOXA1 expression level data were classified into a group with low FOXA1 expression and a group with high FOXA1 expression. The group with low FOXA1 expression included 125 HCC samples, and the group with high FOXA1 expression included 240 HCC samples (the follow-up time/prognosis of 9 samples were missing). The survival package in R software was used for prognostic analysis, and the results showed that FOXA1 expression was significantly correlated with the disease-specific survival and progression-free interval of HCC patients (Figure 4(a)). The disease-specific survival of patients with low FOXA1 expression was significantly better than that of patients with high FOXA1 expression (*P* = 0.044), and the progression-free interval of patients with low FOXA1 expression was also significantly better than that of patients with high FOXA1 expression (*P* = 0.01). Receiver operating characteristic (ROC) curve analysis confirmed that FOXA1 had a high diagnostic value for HCC (AUC = 0.686) (Figure 4(b)). Based on the median FOXA1 expression in tissues, the samples were classified into a group with high FOXA1 expression and a group with low FOXA1 expression (187 cases in each group). An analysis of the clinicopathological characteristics of HCC patients in the group with high FOXA1 expression group and the group with low FOXA1 expression revealed that the group with high FOXA1 expression had a lower proportion of male patients, a lower age of onset, and higher AFP expression than the group with low FOXA1 expression, but no significant differences in the race, cancer stage, or vascular invasion were found between the two groups (*P* > 0.05, Table 1). TCGA dataset was classified into high-expression and low-expression groups based on the FOXA1 expression level, and DEGs were selected for pathway enrichment analysis. High FOXA1 expression was mainly associated with extracellular matrix organization, cancer, and mitosis (Figure 4(c)).

### 3.3. The Expression of FOXA1 Protein in HCC Tissues Was Detected by IHC

**Figure 5:**
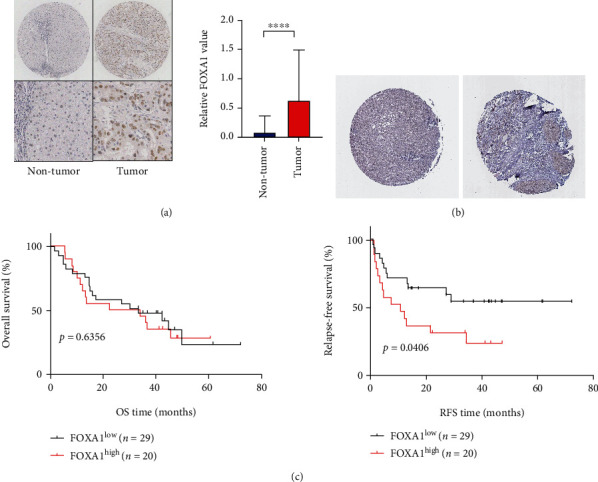
Expression of FOXA1 protein in HCC tissues detected by IHC. (a) IHC staining of an HCC tissue microarray with antibodies specific to FOXA1 (left). Protein levels of FOXA1 in HCC and adjacent nontumor tissues (right). (b) IHC showed the FOXA1 protein levels in HCC and adjacent nontumor tissues based on HPA data. (c) Kaplan–Meier analysis of overall survival and relapse-free survival stratified by the FOXA1 protein expression level. ^∗∗∗∗^: *P* < 0.001.

IHC was performed to detect the FOXA1 protein expression levels in 49 HCC tissues and paired paracancerous tissue samples, and the results showed that positive FOXA1 expression was mainly located in the nucleus of the HCC cells (Figure 5(a)). FOXA1 protein was highly expressed in HCC tissues, with a positive expression rate of 40.8% (20/49), whereas FOXA1 protein was expressed at low levels in paracancerous tissues, with a positive expression rate of 8.2% (4/49); this difference was significant (Figure 5(a), *P* < 0.001). FOXA1 protein expression in HCC tissues was not significantly correlated with sex, age, grade, clinical stage, or other factors (*P* > 0.05) (Table 2). Based on the IHC score, the HCC samples were classified into two groups, a group with high HCC expression and a group with low HCC expression, survival curves were drawn, and the survival rates of the two groups were compared. The prognostic analysis showed that HCC patients with low FOXA1 expression had a longer survival time without recurrence (Figure 5(c), *P* = 0.0406). Although no difference in the overall survival time was found between patients with high FOXA1 expression and patients with low FOXA1 expression (Figure 5(c), *P* = 0.6356), the overall survival curve of the patients with low FOXA1 expression was higher than that of the patients with low FOXA1 expression. The protein level of FOXA1 in HCC patients was verified using the Human Protein Atlas (HPA) database, and the results showed that the FOXA1 protein expression level in HCC tissues was significantly higher than that in normal tissues (Figure 5(b)).

### 3.4. Construction of FOXA1-Overexpressing Cell Lines

**Figure 6:**
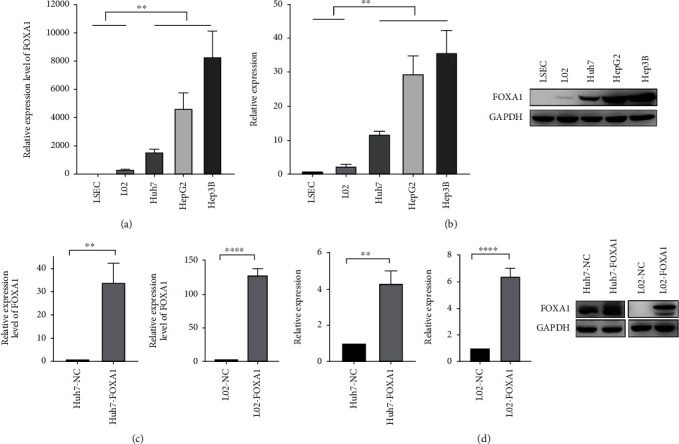
Construction of FOXA1-overexpressing cell lines. (a) Differential mRNA expression of FOXA1 in HCC cell lines (Huh7, HepG2, and Hep3B cells) compared with immortalized normal human hepatic LSEC and L02 cell lines. (b) Results from the Western blot analysis: differential expression of FOXA1 in HCC cell lines and immortalized normal human hepatic cell lines. (c) RT–qPCR assays were performed to detect FOXA1 expression in FOXA1-overexpressing and no-load cell lines. (d) Western blotting was performed to detect FOXA1 protein expression in FOXA1-overexpressing and no-load cell lines. ^∗^: *P* < 0.05; ^∗∗^: *P* < 0.01; and ^∗∗∗^/^∗∗∗∗^: *P* < 0.001.

The expression of FOXA1 in normal liver cell lines and HCC cell lines was detected by RT–qPCR and Western blotting. The results showed that FOXA1 expression in HCC cell lines was significantly higher than that in immortalized human liver cell lines (*P* < 0.01) (Figures 6(a) and 6(b)). In addition, FOXA1 was also expressed at different levels in different HCC cell lines, and the highest and lowest expression levels were found in Hep3B and Huh7 cells, respectively. The mRNA expression level of FOXA1 in Hep3B cells was 5.2-fold higher than that in Huh7 cells, and the expression in HepG2 cells was 2.9-fold higher than that in Huh7 cells. The protein expression level of FOXA1 in Hep3B cells was 3-fold higher than that in Huh7 cells, and the expression in HepG2 cells was 2.5-fold higher than that in Huh7 cells. To investigate the effect of FOXA1 on the malignant phenotype of HCC cell lines and immortalized human liver cell lines, the FOXA1-overexpressing construct and no-load plasmids were transfected into the Huh7 cell line and L02 immortalized human liver cell line with low FOXA1 expression. The mRNA and protein expression levels of FOXA1 in the Huh7 and L02 cell lines were detected by real-time PCR and Western blotting assays, and the expression levels of FOXA1 were both significantly increased, indicating the successful overexpression of FOXA1 in Huh7 cells (Figures 6(c) and 6(d)).

### 3.5. FOXA1 Enhances the Proliferation of Cancer Cells In Vitro and In Vivo

**Figure 7:**
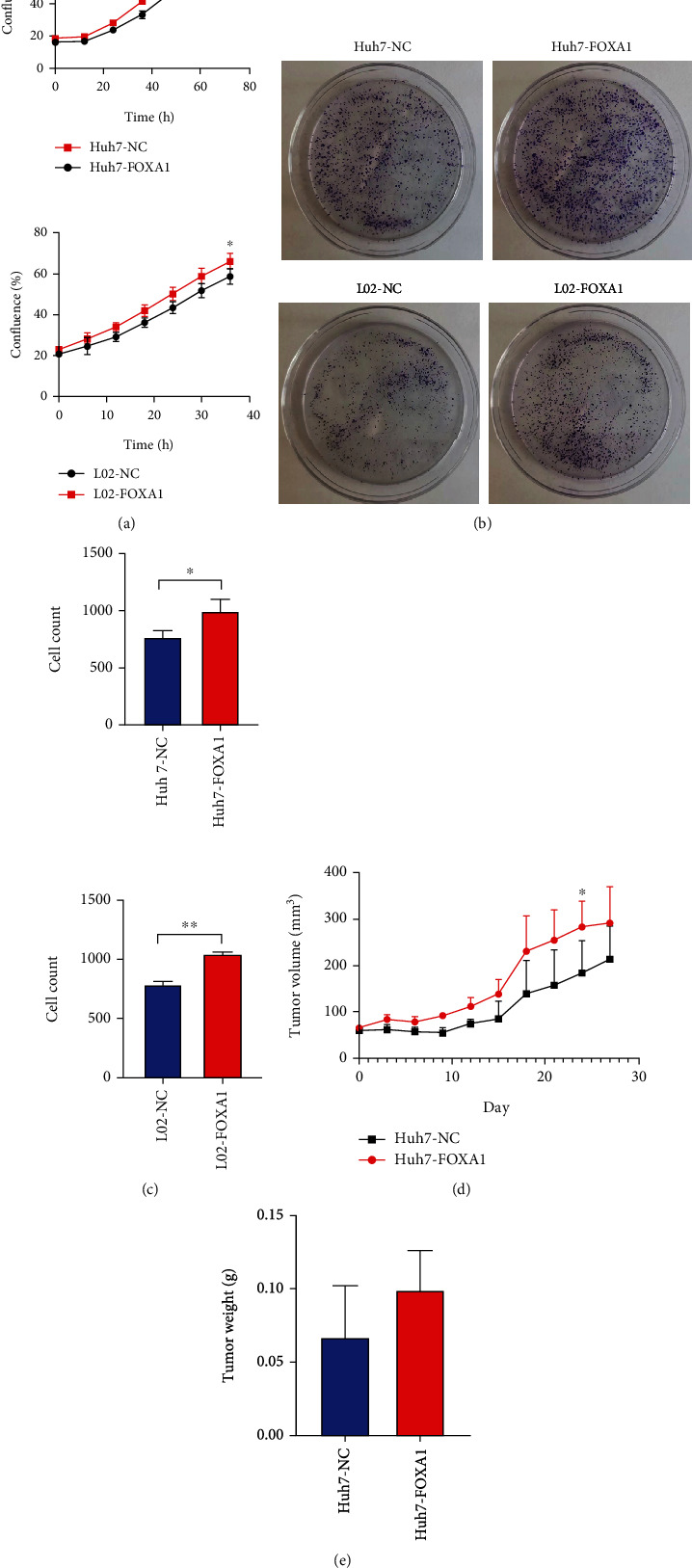
FOXA1 enhances the proliferation of cancer cells in vitro and in vivo. (a) The growth rates of the FOXA1-overexpressing and no-load Huh7 and L02 cell lines were compared. (b) A colony formation assay was performed with Huh7 and L02 cells. (c) The results from the statistical analysis related to colony formation are shown. (d) The tumor volumes were measured during their growth process using a Vernier caliper and calculated with the formula (length × width^2^)/2. (e) The tumor weights were measured after dissection.

FOXA1-overexpressing and no-load cells were seeded on 96-well plates with medium and placed in a live cell monitoring instrument for dynamic photography and observation. The results of the proliferation experiment showed that the FOXA1-overexpressing cells grew significantly faster than the no-load group (Figure 7(a)). These results indicated that the proliferative ability of Huh7 and L02 cells was significantly improved after the overexpression of FOXA1.The results of the colony formation assay showed that FOXA1 significantly facilitated the colony formation ability of Huh7 and L02 cells (Figures 7(b) and 7(c)). Moreover, to detect the functions of FOXA1 in cancer cells in vivo, Huh7 cells with FOXA1-overexpressing and no-load plasmids were hypodermically injected into NTG mice. The results indicated that FOXA1 overexpression promoted tumor growth in terms of tumor weight and volume (Figures 7(d) and 7(e); Figure S1).

### 3.6. Effects of FOXA1 Overexpression on Cell Migration

**Figure 8:**
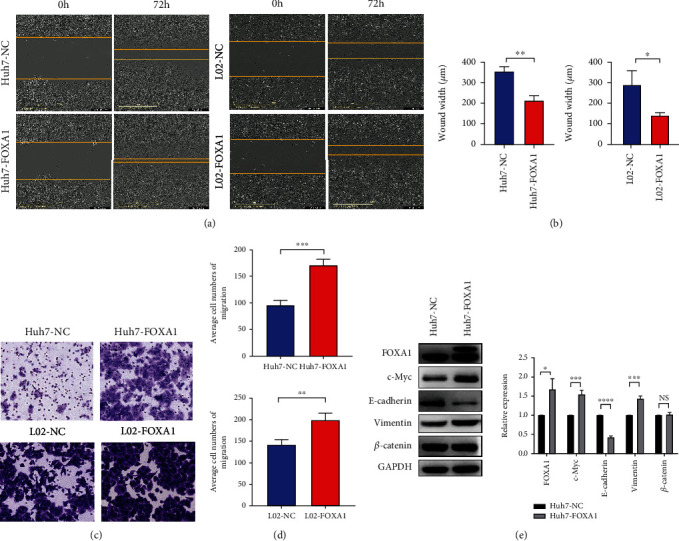
Effects of FOXA1 overexpression on cell migration. (a) Overexpression of FOXA1 in the Huh7 and L02 cell lines led to higher motility in the wound healing assay compared with that of the control cells. (b) The results from a statistical analysis of the findings from the wound healing assay are shown. (c) The Transwell method was used to detect the migratory ability of FOXA1-overexpressing Huh7 and L02 cell lines. Magnification: 10×. (d) Statistical analysis of the results from the Transwell migration assays. (e) The expression levels of E-cadherin, Vimentin, c-Myc, and *β*-catenin in FOXA1-overexpressing and no-load Huh7 cells lines were detected by Western blot analysis. ^∗^: *P* < 0.05; ^∗∗^: *P* < 0.01; and ^∗∗∗^/^∗∗∗∗^: *P* < 0.001.

To exploit the role of FOXA1 in hepatocellular carcinoma cells, we determined the effect of FOXA1 on cell migration by wound healing and Transwell migration assays. The results of the wound healing assay showed that the wound width in the FOXA1-overexpressing group was smaller than that in the no-load group (Figures 8(a) and 8(b); Figure S2), indicating that the migration ability of Huh7 and L02 cells was significantly improved after the overexpression of FOXA1. Transwell migration experiments showed that the migration ability of Huh7 and L02 cells overexpressing FOXA1 was significantly enhanced (*P* < 0.01) (Figures 8(c) and 8(d)). To explore the signaling pathway through which FOXA1 plays a role, we detected the changes in the invasion and metastasis of marker proteins and c-Myc proteins by Western blot assays. The results showed that FOXA1 overexpression inhibited the expression of E-cadherin in Huh7 cells, enhanced the expression of Vimentin and c-Myc protein, and did not affect the expression of *β*-catenin protein (Figure 8(e)).

## 4. Discussion

As one of the malignant tumors in the digestive system, HCC is more common in the Asian population [21]. Despite the increasing number of treatments for HCC, the efficacy of these therapies remains not ideal. Therefore, an improved elucidation of the molecular mechanism of HCC and the identification of new key targets are urgently needed.

The FOXA1 gene is located at the 14q21.1 site on the human chromosome, and its structure includes an N-terminal transcriptional activation domain, the FOX domain binding to DNA in the middle, and the C-terminal and H3/H4 histone-related transcriptional activation binding domains [22]. As a pioneer TF, FOXA1 can replace histone H1 due to its similar structure to maintain other nucleosomes for TF binding and the activation of downstream gene expression [23], which are crucial processes for the normal development of multiple endoderm-derived organs [24]. Studies have shown that FOXA1 is expressed in a variety of cancers, but its role varies in different tumors [25–28]. In addition, FOXA1 has been found to interact directly with androgen receptors and plays a central role in mediating AR-driven oncogenesis [24]. In a study of HCC, Wang et al. found that the long noncoding RNA (lncRNA) McM3ap-as1 directly binds to microRNA miR-194-5p and acts as a competitive endogenous RNA to regulate FOXA1 expression in HCC cells, which results in promotion of the occurrence and development of HCC [29]. Yuan et al. found that FOXA1 is the target of miR-212-3p and that FOXA1 exerts its biological function by regulating the expression of AGR2. FOXA1 is highly expressed in HCC cells, and this TF can promote the proliferation and inhibit the apoptosis of HepG2 cells [30]. In summary, these studies suggest that FOXA1 may play an important role in the development and progression of HCC.

In this study, we obtained 2271 common DEGs, including 1332 common upregulated genes and 939 common downregulated genes, through an analysis of the DEGs in HCC tissues and paracancerous tissues in the GSE121248 and GSE62232 datasets downloaded from the GEO database. The key genes were screened using the STRING database and visualized using Cytoscape software. A literature review revealed that FOXA1 is an essential TF in liver development, and we thus selected FOXA1 for further analysis and verification. An analysis of transcriptome data of 374 HCC samples and 50 paracancerous tissue samples obtained from the TIMER and TCGA databases revealed that the FOXA1 expression level was significantly higher in HCC tissue samples. By performing grouping and prognostic analyses of transcriptomic data in TCGA database, we found that the progression-free interval and disease-specific survival of patients with low FOXA1 expression were significantly better than those of patients with high FOXA1 expression. ROC curves were used to assess the prognostic capacity of FOXA1. The results showed that FOXA1 had a high diagnostic value (AUC = 0.686). TCGA data were then classified into high-expression and low-expression groups according to FOXA1 expression, and DEGs were selected for pathway enrichment analysis. High expression of FOXA1 was mainly related to extracellular matrix organization, cancer, and the M phase, among other pathways. The expression level of FOXA1 protein in HCC tissues and paracancerous tissue samples was detected by IHC, and the results showed that the FOXA1 protein was significantly overexpressed in HCC tissues. A prognostic analysis showed that HCC patients with low FOXA1 expression had longer survival times without recurrence.

The GSEA results suggest that FOXA1 promotes the progression of liver cancer by promoting the proliferation and metastasis of HCC cells. We wanted to test this hypothesis by observing the effect of FOXA1 overexpression on cell proliferation and migration.

First, RT–qPCR and Western blot experiments showed that FOXA1 expression in normal liver cell lines was significantly different from that in HCC cell lines, and FOXA1 expression in HCC cell lines was higher than that in immortalized human liver cell lines. FOXA1 was then overexpressed in the Huh7 and L02 cell lines. Proliferation, colony formation, wound healing, and Transwell assays verified that FOXA1 overexpression affected the malignant phenotype of the Huh7 and L02 cell lines and enhanced the proliferation and migration abilities of the Huh7 and L02 cells were significantly enhanced after FOXA1 overexpression. Moreover, we generated a xenograft model in NTG mice by subcutaneous injection with HCC cells, and the results showed that the overexpression of FOXA1 in xenografted mice accelerated the growth of HCC.

In addition, previous studies have verified the role of FOXA1 in tumorigenesis by inhibiting the expression of FOXA1. Gan et al. found that FOXA1 silencing can suppress liver cancer stem cell proliferation and regulate cell apoptosis [31]. A study of nonsmall cell lung cancer found that FOXA1 siRNA transfection caused G0/G1 phase cell cycle arrest and reduced the invasion, migration, and proliferation abilities of A549 cells [32]. Imamura et al. found that depletion of FOXA1 in a prostate cancer cell line using small interfering RNA significantly inhibits AR activity, leads to cell growth suppression, and induces G0/G1 arrest [33]. Other studies have found that FOXA1 suppresses breast cancer cell growth and inhibits apoptosis [34]. A previous study of endometrial cancer found that FOXA1 knockdown reduces the rate of tumor growth in an in vivo xenograft model [35].

In summary, this study found that FOXA1 protein was significantly overexpressed in HCC cell lines and tissues of HCC patients, and high FOXA1 expression in HCC tissues was significantly correlated with poor prognosis in patients. We also validated the effect of FOXA1 on promoting the proliferation and migration of HCC cells. FOXA1 is a key molecule in the occurrence and development of malignant tumors, which suggests that FOXA1 may play an oncogenic role in hepatocellular carcinoma. Further study of the TF FOXA1 in HCC can increasingly clarify its role in the occurrence and development of HCC, and FOXA1 will likely become a new therapeutic target in HCC.

## Figures and Tables

**Table 1:** Clinicopathological parameters of 374 hepatocellular carcinoma patients in TCGA database.

Characteristic	Low expression of FOXA1	High expression of FOXA1	*P*
*N*	187	187	
Gender, *n* (%)			<0.001
Female	42 (11.2%)	79 (21.1%)	
Male	145 (38.8%)	108 (28.9%)	
Race, *n* (%)			0.936
Asian	81 (22.4%)	79 (21.8%)	
Black or African American	9 (2.5%)	8 (2.2%)	
White	91 (25.1%)	94 (26%)	
Age, *n* (%)			0.011
≤60	76 (20.4%)	101 (27.1%)	
>60	111 (29.8%)	85 (22.8%)	
AFP (ng/ml), *n* (%)			0.043
≤400	119 (42.5%)	96 (34.3%)	
>400	26 (9.3%)	39 (13.9%)	
T stage, *n* (%)			0.109
T1	102 (27.5%)	81 (21.8%)	
T2	42 (11.3%)	53 (14.3%)	
T3	37 (10%)	43 (11.6%)	
T4	4 (1.1%)	9 (2.4%)	
N stage, *n* (%)			1.000
N0	132 (51.2%)	122 (47.3%)	
N1	2 (0.8%)	2 (0.8%)	
M stage, *n* (%)			1.000
M0	140 (51.5%)	128 (47.1%)	
M1	2 (0.7%)	2 (0.7%)	
Pathologic stage, *n* (%)			0.280
Stage I	96 (27.4%)	77 (22%)	
Stage II	42 (12%)	45 (12.9%)	
Stage III	37 (10.6%)	48 (13.7%)	
Stage IV	3 (0.9%)	2 (0.6%)	
Vascular invasion, *n* (%)			0.452
No	109 (34.3%)	99 (31.1%)	
Yes	52 (16.4%)	58 (18.2%)	

**Table 2:** Relationship between FOXA1 protein expression levels in HCC tissues and clinicopathological parameters.

Characteristic	Number	FOXA1		*χ* ^2^	*P*
		Low expression	High expression		
Gender					
Male	45	28	17	2.107	0.147
Female	4	1	3		
Age (years)					
>60	5	4	1	0.999	0.318
≤60	44	25	19		
Tissue					
Para-carcinoma tissue	49	45	4	14.126	*P* < 0.0001
HCC tissue	49	20	29		
Grade					
II	22	15	7	2.565	0.277
II-III	9	6	3		
III	18	8	10		
TNM stage					
I-II	25	14	11	0.214	0.644
III-IV	24	15	9		
Tumor diameter (cm)					
≥3	36	22	14	0.209	0.648
<3	13	7	6		
Tumor numbers^a^					
≥2	13	10	3	3.255	0.196
<2	35	18	17		
Vascular tumor emboli^a^					
Yes	10	4	6	2.202	0.138
No	38	25	13		

Note: ^a^this information is missing.

## Data Availability

The GSE121248 and GSE62232 mRNA expression datasets were downloaded from the Integrated Gene Expression Omnibus (GEO) (http://www.ncbi.nlm.nih.gov/geo). Transcriptome data from 374 samples of HCC tissues and 50 samples of normal tissues and patients' clinical information were downloaded from The Cancer Genome Atlas database (https://portal.gdc.cancer.gov/).

## Abbreviations

HCC: Hepatocellular carcinoma
CCA: Cholangiocarcinoma
TFs: Transcription factors
FOXA1: Forkhead box A1
FHD: Forkhead box domain
AR: Androgen receptor
ER: Estrogen receptor
GEO: Gene Expression Omnibus
TCGA: The Cancer Genome Atlas
IHC: Immunohistochemistry
GO: Gene Ontology
KEGG: Kyoto Encyclopedia of Genes and Genomes
HPA: Human Protein Atlas
TIMER: Tumor immune estimation resource
ROC: Receiver operating characteristic
LUAD: Lung adenocarcinoma
LUSC: Lung squamous cell carcinoma
CESC: Cervical squamous cell carcinoma and cervical adenocarcinoma.

## Supplementary Materials

Supplementary Materials Figure S1: tumor xenograft model. (a) Xenograft tumors retrieved from animals. (b) The original images of the mouse. Figure S2: overexpression of FOXA1 in the Huh7 and L02 cell lines led to higher motility in the wound healing assay compared with that of the control cells.
