# Supplementary material for: Elevated FOXA1 Expression Indicates Poor Prognosis in Liver Cancer due to Its Effects on Cell Proliferation and Metastasis
Source: Dis Markers. 2022 Aug 5;2022:3317315. doi: 10.1155/2022/3317315 (PMC9374549; doi:10.1155/2022/3317315)
Supplement: Supplementary Materials — Figure S1: tumor xenograft model. (a) Xenograft tumors retrieved from animals. (b) The original images of the mouse. Figure S2: overexpression of FOXA1 in the Huh7 and L02 cell lines led to higher motility in the wound healing assay compared with that of the control cells. [file 3317315.f1.docx]

**Supplementary Materials for**

**Elevated FOXA1 expression indicates poor prognosis in liver cancer** **due to its effects on cell** **proliferation and metastasis**

Zhenrong Liu^1^, Yaru Wang^1^, Zulihumaer Aizimuaji^1^, Sheng Ma^1^, Ting Xiao(🖂)^1^

^1^State Key Laboratory of Molecular Oncology, Department of Etiology and Carcinogenesis, National Cancer Center/National Clinical Research Center for Cancer/Cancer Hospital, Chinese Academy of Medical Sciences and Peking Union Medical College, Beijing, 100021, China.

**Correspondence to:**

Ting Xiao, MD, State Key Laboratory of Molecular Oncology, Department of Etiology and Carcinogenesis, National Cancer Center/National Clinical Research Center for Cancer/Cancer Hospital, Chinese Academy of Medical Sciences and Peking Union Medical College, Beijing, 100021, China. Tel: +86 10 87788726; Fax: 87707363; E mail: xiaot@cicams.ac.cn.

**This PDF file includes:**

Supplementary Text

FigureS1 and FigureS2


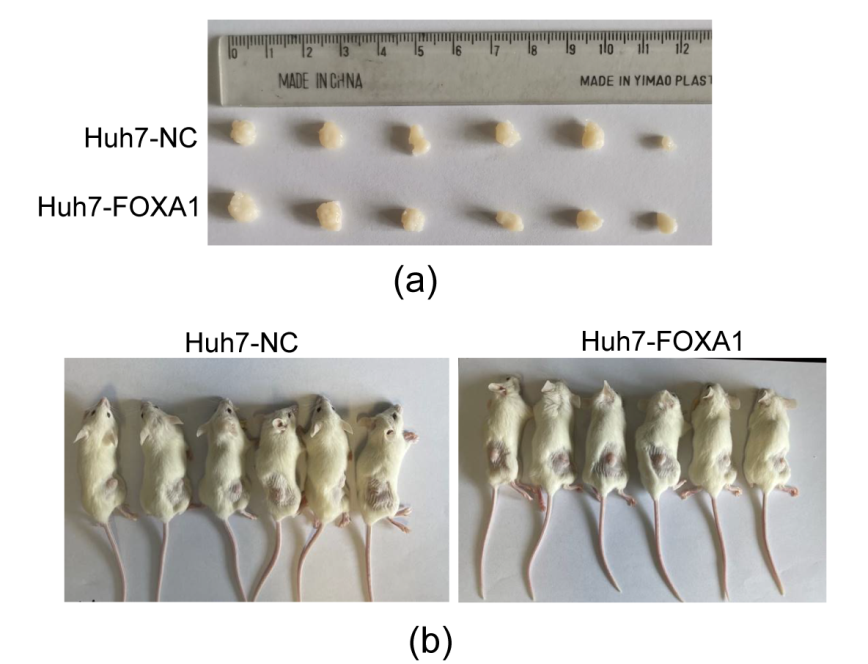


FigureS1. Tumor xenograft model (a) Xenograft tumors retrieved from animals. (b) The original images of the mouse.


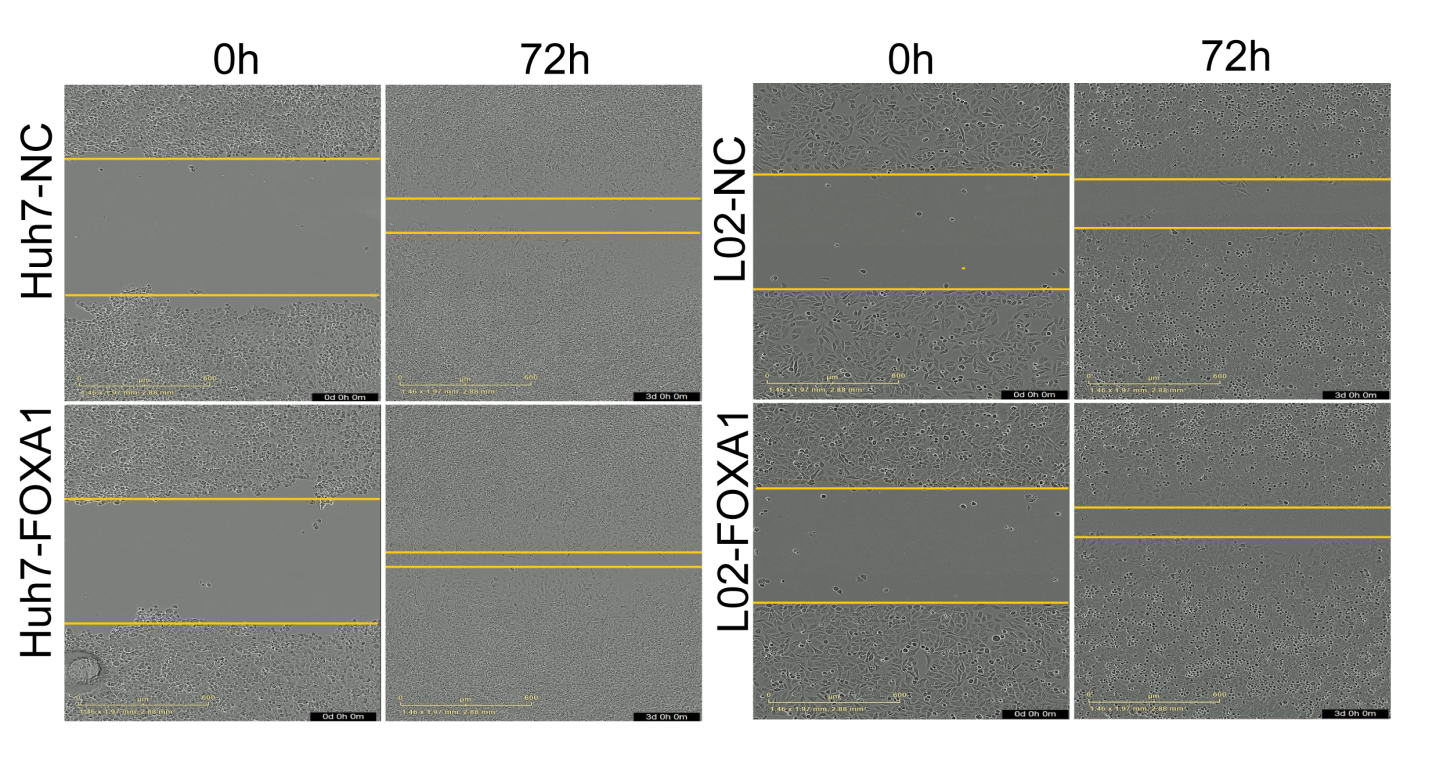


FigureS2 Overexpression of FOXA1 in the Huh7 and L02 cell lines led to higher motility in the wound healing assay compared with that of the control cells.
